# Operationalizing racialized exposures in historical research on anti-Asian racism and health: a comparison of two methods

**DOI:** 10.3389/fpubh.2023.983434

**Published:** 2023-07-06

**Authors:** Marie Kaniecki, Nicole Louise Novak, Sarah Gao, Sioban Harlow, Alexandra Minna Stern

**Affiliations:** ^1^University of Michigan, Ann Arbor, MI, United States; ^2^University of California, Los Angeles, Los Angeles, CA, United States; ^3^College of Public Health, The University of Iowa, Iowa City, IA, United States; ^4^Public Policy Center, The University of Iowa, Iowa City, IA, United States; ^5^Harvard Center for Population and Development Studies, School of Public Health, Harvard University, Cambridge, MA, United States; ^6^School of Public Health, University of Michigan, Ann Arbor, MI, United States

**Keywords:** United States census, Asian Americans, racialization, surname, history, structural racism, misclassification and its error, sensitivity and specificity

## Abstract

**Background:**

Addressing contemporary anti-Asian racism and its impacts on health requires understanding its historical roots, including discriminatory restrictions on immigration, citizenship, and land ownership. Archival secondary data such as historical census records provide opportunities to quantitatively analyze structural dynamics that affect the health of Asian immigrants and Asian Americans. Census data overcome weaknesses of other data sources, such as small sample size and aggregation of Asian subgroups. This article explores the strengths and limitations of early twentieth-century census data for understanding Asian Americans and structural racism.

**Methods:**

We used California census data from three decennial census spanning 1920–1940 to compare two criteria for identifying Asian Americans: census racial categories and Asian surname lists (Chinese, Indian, Japanese, Korean, and Filipino) that have been validated in contemporary population data. This paper examines the sensitivity and specificity of surname classification compared to census-designated “color or race” at the population level.

**Results:**

Surname criteria were found to be highly specific, with each of the five surname lists having a specificity of over 99% for all three census years. The Chinese surname list had the highest sensitivity (ranging from 0.60–0.67 across census years), followed by the Indian (0.54–0.61) and Japanese (0.51–0.62) surname lists. Sensitivity was much lower for Korean (0.40–0.45) and Filipino (0.10–0.21) surnames. With the exception of Indian surnames, the sensitivity values of surname criteria were lower for the 1920–1940 census data than those reported for the 1990 census. The extent of the difference in sensitivity and trends across census years vary by subgroup.

**Discussion:**

Surname criteria may have lower sensitivity in detecting Asian subgroups in historical data as opposed to contemporary data as enumeration procedures for Asians have changed across time. We examine how the conflation of race, ethnicity, and nationality in the census could contribute to low sensitivity of surname classification compared to census-designated “color or race.” These results can guide decisions when operationalizing race in the context of specific research questions, thus promoting historical quantitative study of Asian American experiences. Furthermore, these results stress the need to situate measures of race and racism in their specific historical context.

## Introduction

### Why is historical research important for discussing anti-Asian racism and health?

Scholars have consistently identified gaps in the literature concerning Asian-American health (1, 2) and associations between racial discrimination and health for Asian Americans (3). Calls to address these gaps have taken on new urgency in the United States, where scholars and activists have identified a rise of anti-Asian discrimination and hate crimes during the global COVID-19 pandemic (4–8). This surge in discrimination is not a new phenomenon; it exemplifies the racist association of Asian bodies with disease that originated on the West Coast of the United States in the mid-19th century (9, 10). As many other scholars have attested, addressing contemporary racism and its impacts on health requires understanding its historical roots (11–13).

This paper does not examine a specific health outcome, rather expands the discussion on methods and assumptions critical to historical health research. We explore the strengths and limitations of two different approaches to operationalizing racialized exposures, surname matching and enumerator racial classification, using historical census data from 1920, 1930, and 1940 (14) as a case study for Asian Americans.

Operationalizing racism in different time periods requires carefully considering processes of racialization and the specific origins of different historical data sources (13, 15, 16). Archival secondary data such as historical census records lend themselves to quantitative analysis of structural dynamics that affect the health of Asian immigrants and Asian Americans. Historical census data overcome common weaknesses of other data sources, such as small sample size and aggregation of Asian sub-groups. However, white supremacist and eugenic ideologies informed census enumeration procedures (9, 10, 17), raising questions about the validity of census racial measures over time. This presents challenges when operationalizing racialized exposures of Asian Americans using historical census data.

### A brief outline

The remaining three subsections of our introduction further establish the theoretical groundwork for the comparison of surname matching and enumerator racial classification, synthesizing the varied and sometimes conflicting literature definitions of race and related terms, detailing challenges specific to quantitative historical research on Asian Americans, and outlining how racial classification and surname matching criteria operationalize racism or racialized exposures.

The methods section describes the generation of the census datasets and surname lists used in the analysis, how well our populations of interest meet underlying methodological assumptions for application of surname criteria, the definition of validity measures calculated, and the analytical process.

In the results section, we first present demographics and descriptive statistics of the three census populations, then tabulate the validity statistics we calculated alongside those calculated by Lauderdale and Kestenbaum with 1990 census data, and finally describe individual results for sensitivity, specificity, and PPV in more detail.

Our discussion section compares our results to the validity measures for the 1990 census, contextualizes our validity measures for our populations of interest for analytical applications, offers possible explanations for lower-than-expected validity measures and the disagreement between the two classification methods, and connects our questions about the validity of these methods to literature examining similar questions for other populations or in other time periods.

Finally, our conclusion connects our findings back to the broader research implications of our results and highlights the importance of these types of research questions to contemporary health outcomes.

### What do we mean when we talk about race?

Many public health studies use racial classification as a proxy for racialized exposures. Unfortunately, many of these same studies fail to provide adequate methodological explanation of how race is conceptualized and operationalized when included in a study. In fact, a systematic review that examined a stratified sampling of publications from five major epidemiology journals from 1995 to 2018 found that out of 329 studies including data on individuals’ race and/or ethnicity, only four studies provided even a working definition of this construct and the majority of studies were unclear about how they measured race and/or ethnicity (16). As Roberts and Adkins-Jackson et al. assert, researchers who do not sufficiently illustrate their basic conceptualization and operationalization of race in their studies end up “filter[ing] out the impact of race” (18) or reifying “erroneous assumptions about the biological differences between racialized groups” (19). Furthermore, Adkins-Jackson et al. identify problems associated with using race as a variable in place of racism (19) and a growing body of literature investigates more salient methods to measure and analyze racialized exposures and racism at structural, institutional, and interpersonal levels (19–25). Public health researchers conducting prospective studies should strongly consider incorporating these more nuanced methods into their study design, data collection, and analysis (16, 19–25).

However, racial classifications remain an important if imperfect proxy (18), especially when conducting retrospective and historical research. Operationalizing racism in a meaningful way by using existing classification data requires a thorough understanding of several concepts related to and often conflated with race. Ethnicity, national origin, and ancestry are often incorrectly used as euphemisms for race (18, 20, 26). Factors such as immigration (9, 10) and the collapsing of national or ethnic categories (27, 28) require special consideration in the context of Asian racial formation in the United States. The remainder of this subsection outlines the conceptualization of race and related terms that we employ in this study, in line with recommendations for epidemiology and other health fields (16).

Race is now widely recognized as a social and political construct rather than an inherent, biologically-determined characteristic (17–19, 29, 30). Throughout history, varying physical characteristics have been ascribed social and political meaning to enforce hierarchies of power, with whiteness situated at the top (18, 29, 31). This racialization of bodies is highly context specific (10, 29), developing and changing over time and across geographic location in a process Omi and Winant call racial formation (29). Racist scientific rhetoric helped maintain unequal and exploitive power structures, using flawed methodologies developed in the fields of phrenology and eugenics to assert that race was a measure of innate biological superiority or inferiority (17). Rejecting the biologic basis for race does not mean it is immaterial in the realm of health (18, 29). Health inequities among racial groups stem from the social consequences of racialization, impacting health through biological mechanisms such as access to health resources and stress associated with institutional and interpersonal racism (18).

Ethnicity and race are not only conflated in meaning, but are often combined into a single term, “race/ethnicity” (20). In some ways a reaction to the externally ascribed nature of race (29), ethnicity is typically conceptualized as self-selected membership in a cultural group (20, 22, 29). As with race, it is informed by a mix of nationality, ancestral national origin, and physical appearance (20, 29, 32). More nuanced definitions of ethnicity have incorporated a relational dimension, acknowledging external hierarchical influences on cultural identity and ethnicity (20). Importantly, ethnicities can also function as subcategories of racial groups (20). For example, Chinese-Americans, Indian-Americans, Japanese-Americans, Korean-Americans, and Filipino-Americans (along with numerous other ethnic groups) would comprise the pan-ethnic racial category of Asian-American (27, 29). The current United States census definition of ethnicity incorporates the basic tenets of the cultural definition of ethnicity described above, but differs markedly in that it only delineates two ethnic groups, Hispanic and non-Hispanic (33, 34), and allows those with Hispanic ethnicity to fall into any other racial category (21).

National origin refers to a person’s country of birth (20, 26). Nationality is sometimes used equivalently, but it constitutes a legal status associated with naturalization (10, 35) and thus may also refer to a person’s country of citizenship after migration. Asian ethnic groups are often condensed in the United States context post-immigration to adhere to national origin boundaries. However, this equivalency of ethnicity and national origin constitutes erasure of multi-ethnic states of origin, consolidating culturally diverse populations (36, 37) into single American ethnic groups. For example, the Chinese population is made up of 56 officially recognized ethnic groups and many additional ethnic groups that do not have official government recognition (37). Yet ethnic groups within China such as Han, Zhuang, and Hui (37) rarely translate into hyphenated American identities in the way of Chinese-American identity. Furthermore, equating ethnicity and national origin does not account for international migration of previous generations (18), changes in state borders over time (10, 18), and the existence of stateless peoples (10). Ngai argues that the supposedly objective characteristic of “national origin” had differential importance in defining social hierarchies for whites and non-whites when it was first created and defined in the early 20th century. Non-whites were grouped together mainly by race with national origin de-emphasized, whereas the foregrounding of national origins for Europeans served to selectively exclude “undesirable” European immigrants under the Immigration Act of 1924 (10).

Parental national origin or nationality informs ethnic identity and constitutes a component of ancestry. Ancestry typically denotes a person’s broadly defined heritage or descent (22). More specifically, ancestry can refer to either ancestral national or cultural origin (20) or genetic or geographic ancestry (18). Roberts cautions against equating genetic or geographic ancestry with race given that the former concepts are biologically-defined and do not map onto discrete, socially created racial categories. This equivalence only serves to reify problematic conceptualizations of races as natural divisions among humans. Ancestry, when applied correctly, is a highly individual characteristic rather than a homogenous group identity. It has the added conceptual advantage of allowing mixed ancestral nationalities and not needing the delineation of mutually exclusive categories (18).

Immigration plays a key role in the racialization of Asian immigrants and Asian Americans alike. The racial triangulation theory posits that racialization occurs along two axes: inferior–superior and foreigner-insider (38). Public health narratives and xenophobic, racist rhetoric consistently portrayed Asian populations as unassimilable, perpetual foreigners, creating what Ngai calls “alien citizens” (10). Despite the demonstrated history of racializing immigrants on the basis of their perceived foreignness, research on immigrant populations in the United States tends to prioritize ethnicity at the expense of race. Some researchers have thus called for a “racialization” of immigration studies to incorporate critical race theory (39, 40).

### What challenges do we face when conducting historical quantitative research on Asian Americans?

Beyond the complexity in defining race and related concepts, historical quantitative research on Asian Americans is further complicated by methodological challenges and characteristics of available datasets. Historical data sources do not always systematically classify race, but racialization processes were nevertheless salient in the lives of the people in the dataset. For example, our analyses of the racialized implementation of California’s eugenic sterilization program relied on Spanish surname (39) and Asian nativity (40) rather than explicit racial classification. The use of proxies to operationalize a racialized exposure was motivated by the inconsistent collection of race and ethnicity on the institutional forms that comprised our dataset. Historical research is limited to data that have already been collected and often cannot incorporate the many innovative methodologies that prospective survey data collection can facilitate.

As previously discussed, the boundaries of racial categories changed over time (41) and were politically motivated (10, 42). Since researchers operate under their own contemporary racial socialization (15, 16), they could potentially generate research questions predicated on contemporary understandings of race rather than the racial environment of the period of study. Unless rooted in the appropriate historical racial context (13, 15), a flawed underlying conceptual model or inappropriate terminology could bias the research. Similarly, biases introduced into the data at the time of collection must be thoughtfully considered to properly operationalize the information therein. Determining which people to classify as Asian can be difficult if they are described in discriminatory or anachronistic language rather than as Asian or Asian-American. Various national and ethnic Asian subgroups were ascribed a group racial identity of Asian, “Asiatic,” or “Oriental” through the early 20th century (10), but Asian-American only emerged as a named racial identity decades later during the civil rights era (29). In longitudinal studies the racial lexicon and hierarchies of multiple time periods must be taken into account, as well as the processes that produced changes in them over time. Thus, race-related variables may not be directly comparable and could require a harmonization process across time.

Aggregation of different Asian subgroups can statistically mask disparate health outcomes (3, 28). The aggregation of Asian subgroups into a larger Asian-American or Asian American Pacific Islander category can falsely homogenize the experiences of diverse populations. As a pan-ethnic group, Asian Americans in some studies have been shown to have better economic outcomes compared to the overall United States population (43) and similar or better health outcomes compared to white Americans (44). However, aggregation can statistically mask important ethnic differences in residential and occupational segregation (45), economic inequality (43), and health disparities (3, 28, 46). Decisions to aggregate Asian-American subgroups into a single racial category often stems from limitations in data sources, sample size, and feasibility of sampling or analysis rather than from a theoretically salient research question. As is true with contemporary data sources, historical data may lack granular racial or ethnic information. For example, vital statistics compiled by the Los Angeles County Health Department in Annual Health Reports from 1915 to 1926 include only five “racial” categories: White, Black, Mexican, Japanese, and Other. Depression-era reports present vital statistics by the two categories of White and Mexican (47).

### How do census racial classification and surname matching operationalize racism or racialized exposures?

#### Census classification

One approach to operationalizing racialized exposures is by using census racial classification as a proxy for racialized exposures. Self-enumeration did not become the standard until the 1970 census (48); in prior years this variable measures the census enumerator’s external and socially-informed judgment of a person’s racial identity. Census enumeration instructions (see methods and figures for more detail) did not clarify how the enumerator should make this judgment (49–51), implying that elements of such a classification system were commonly known and accepted. Census procedures in the early 20th century did not preclude racial self-identification, but phenotypic observation, residential proximity to ethnic neighborhood enclaves, national origin, parental birthplace, or a combination of those factors likely also influenced the enumerator’s ultimate choice of classification. Thus, this classification method captures the observed and known racial ancestry dimensions of race, with possible influence of self-classification as well (52).

Census racial classification has numerous strengths for examining health at the population-level, whether by itself or in conjunction with other datasets. Health researchers frequently employ the demographic information provided in the census as exposures (neighborhood-level characteristics, socioeconomic status), outcomes (morbidity, mortality, disease incidence), or covariates (age, sex). In addition, census data can provide population-level denominators; stratifying these denominators by race can reveal racial disparities (53).

While self-identification of race is currently the standard in federal data collection (54), Kaplan and Bennet argue that “self-report may not fully capture the effects of discrimination, which is more likely to be based on observers’ perceptions than on self-perception” (55) and Cobb et al. illustrate how “socially-assigned” dimensions of race shape health disparities (56). In 1970, the Census Bureau compared self-identified race with enumerator observed race. Although agreement was fairly high between the two measures for white and black populations (>95% agreement), a much lower level of agreement (73%) was found for Asian and Native American populations (48). The racialized nature of census enumeration means these historical enumerator categorizations may more directly capture some elements of structural racism beyond the proxy-level. Ironically, the messy conflation of race, ethnicity, national origin, and even religion (Asian Indians were called “Hindus” regardless of religion) may constitute a relative strength of early twentieth-century census data: rather than a pan-ethnic “Asian” category, the census documented multiple separate “races” (Chinese, Japanese, etc.), which provides disaggregated data for what today would be considered Asian-American subgroups.

#### Surname criteria

Surname classification has been used to supplement racial or in place of ethnic classification, when racial or ethnic information is absent or limited for many different racial, ethnic, religious and national origin groups. This includes Hispanic or Latino groups (57), people of Arab ancestry (58, 59) European ethnic groups or descendants from specific European countries (60), American Jews (61, 62), South Asians, Asian Americans (63), and others (64, 65). Methods range from matching surnames to existing lists, using surnames in combination with other information such as geographic residence (66), and using hot deck imputation procedures that use surnames in conjunction with racial or ethnic information from similar people in a dataset (67).

Although often used as a proxy for race [capturing elements of the interaction-based observed race and known racial ancestry dimensions of race (52)], it is more accurate to say that surnames may provide insight into ethnicity or ancestral national origin. Historically, many surnames have been distinctive to particular language, culture and ethnic groups. Surname lists are sometimes classified by country of origin (e.g., German, Japanese), but may also be used to distinguish multiple ethnic groups within a particular country, or may identify ethnic groups that span multiple countries. As surnames are typically passed down through families, a person’s surname may provide information about the cultural origin of at least one line of ancestry. Some experimental studies have found that surnames in themselves can lead people to be exposed to racist discrimination (68, 69).

At a population level, surnames can provide a valuable clue about the distribution of ancestral national origin and open up analytic possibilities for data sources that do not have reliable information about race and ethnicity. Surname matching could improve sampling when oversampling or restricting to individuals from a specific ethnic group (70). In addition, when data sources such as the census have inconsistent racial categorizations over time, surnames can provide the needed standardization to classify people for longitudinal research (13).

However, using surnames as a proxy for ascribed race or ethnicity relies on many assumptions, and the usefulness of surname matching will vary across populations and time periods depending on the prevalence of different ancestral groups in the population, enduring legacies of colonization and enslavement, family name practices (e.g., name order, name changes at marriage, patronymic vs. matronymic surnames), rates of marriage between ancestral groups, and other factors.

The use of specific surnames as a proxy for nationality or ethnicity rests upon four main methodological assumptions:

1. Though name order varied by culture, family name was accurately recorded as surname in the source data.
2. The subgroups being classified had low rates of intermarriage with other ethnic or racial groups.
3. Second and subsequent generations have similar surnames to those of first-generation immigrants.
4. The population under study does not contain multiple subgroups with similar surnames.

The validity of surname matching as a proxy for race or ethnicity depends on the extent to which these assumptions are met. Past research, primarily examining Spanish surname criteria, has found that the validity of surname matching criteria varied according to sex (surname criteria had better sensitivity and specificity for men than women) (71, 72); social class (surname criteria had better sensitivity and specificity for people of low socioeconomic status compared to high socioeconomic status) (72, 73); colocation of ethnic groups with similar surnames (e.g., Spanish surname criteria are less valid in populations that also have high concentrations of Filipino, Italian, or Portuguese individuals) (71, 73, 74).

This paper evaluates the validity of contemporary Asian surname matching classifications to enumerator racial classification in the 1920,1930, and 1940 censuses.

## Methods

### Census data

**Figure 1:**
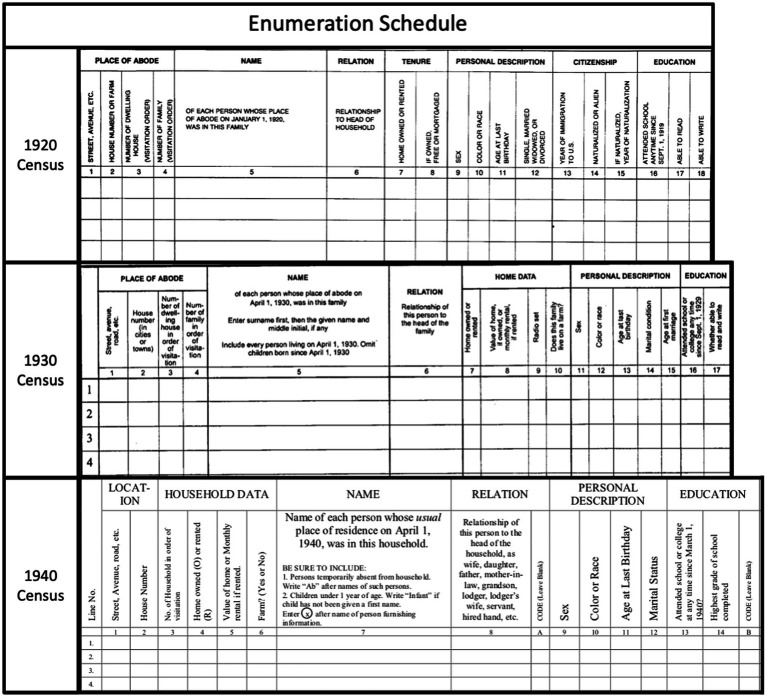
Census population schedules from the 1920, 1930, and 1940 census, courtesy of the U.S. National Archives and Records Administration (NARA).

We used restricted Preliminary Complete Count United States census microdata for 1920, 1930, and 1940 from IPUMS United States (75) which includes individual-level name and demographic information. These datasets were generated by IPUMS USA through collaboration with Ancestry.com. Ancestry.com digitized and transcribed the original handwritten census broadsheets (Figure 1) and IPUMS abstracted these transcriptions into a dataset and performed cleaning and quality checks. More details on the production of these datasets are available elsewhere (76–81). We restricted this analysis to census data from California, a state that has long been home to multiple Asian national origin groups. For the present analysis, we used information on individuals’ sex, age, assigned race, and surname.

During this historical period, census enumerators collected data on handwritten “population schedules.” Instructions to Enumerators documents from 1920, 1930, 1940 lend insight into the norms and standards for data collection during this time (49–51). For each census, enumerators were instructed to approach each dwelling in their assigned enumeration district and record information on each resident of the household. The instructions do not specify how information is to be obtained, whether through respondent self-report or enumerator assessment. The use of interpreters was not encouraged; the 1920 instructions suggest: “In the case of an occasional family that does not speak English or any language which you speak, you can usually get along without the aid of a paid interpreter. If you cannot make the head of the family understand what is wanted, call upon some other member of the family; and if none of the family can understand, then, if possible, obtain the unpaid assistance of some neighbor of the same nationality.” The instructions do describe a process for arranging for interpreter services, but state that “the law does not contemplate that interpreters shall be employed to assist enumerators except in extreme cases” (49). Nearly identical instructions were used in 1930 and 1940 (50, 51).

**Figure 2:**
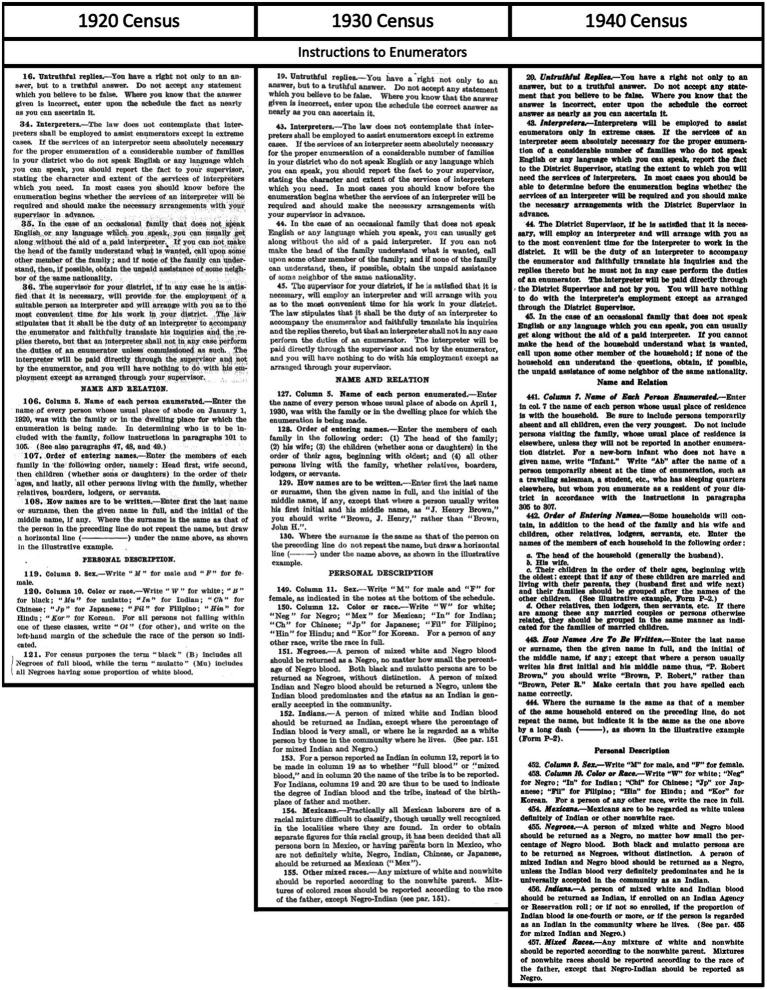
Relevant excerpts from the official Bureau of the Census Instructions to Enumerators in 1920 (49), 1930 (50), and 1940 (51).

Enumerator instructions for sex, age, and place of birth are consistent across the 1920, 1930 and 1940 censuses. Enumerators were instructed to classify sex as “M,” or “F”; age in years as of April 1 of the census year; and place of birth (country or US. State). Some census racial categories (“i.e., Mexican” and the terms used for Black Americans) changed across the three decennial census years in this study, but the categories for people of Asian origin remained consistent: “Chinese, Japanese, Filipino, “Hindu,” and Korean. Figure 2 depicts excerpts from the instructions to enumerators in each of the census years. Instructions varied across census years regarding respondents who did not fit into the specified categories: in 1920, enumerators were instructed to write “Ot” for other and write the respondent’s race in the margin; in 1930 and 1940, they were to “write the race in full.” The 1940 instructions further specified that “Any mixture of white and nonwhite should be reported according to the nonwhite parent. Mixtures of nonwhite races should be reported according to the race of the father, except that Negro-Indian should be reported as Negro.” Instructions on recording surnames are brief: “Enter first the last name or surname, then the given name in full, and the initial of the middle name, if any” (49–51).

### Surname classification

We used Lauderdale and Kestenbaum’s validated surname lists for Asian Indian, Chinese, Filipino, Japanese, Korean, and Vietnamese origin groups, which together include a total of 20,693 surnames (70). These six subgroups constituted approximately 90% of Asian individuals in the dataset used to generate the lists. These lists, originally published in 2000, continue to be applied in multiple disciplines, including political science (82, 83), psychology (83), economics (84), and health research (85–88). The lists of surnames were generated using two data sources: (1) Social Security Administration data on all social security card applicants born outside the United States prior to 1941, using maiden name (as opposed to married surname) for all married women; and (2) data on all persons entitled to social security benefits or enrolled in Medicare, regardless of nativity. Surnames were considered “predictive” if at least 50% of persons with the surname were associated with a specific national origin and” strongly predictive” if at least 75% of persons with the surname were from a specific national origin. The authors generated “conditional” lists for use with surname data that can be restricted to people classified as Asian race, and “unconditional” lists for use in datasets with no race information. To improve the specificity of the Filipino unconditional list, that list excludes all surnames on the Spanish surname list used by the United States Census Bureau. The authors validated the lists against a subfile of 1990 census data, which included a younger population and a higher proportion of United States nativity than the original data sources (70). Lauderdale and Kestenbaum’s lists are methodologically strong compared to other Asian surname lists, in that they have the broadest coverage of Asian ethnic groups and were constructed from a reference population of sufficient size (65).

To classify surnames in the census data, we matched the unconditional, predictive surname list to the surname field in the census data and created indicator variables for individuals whose surname matched with each one of the origin groups. We used the “unconditional” list (as opposed to the list used conditional on classification of Asian race) because census data did not use an overall “Asian” category but rather used Asian subgroups. We used the “predictive” list (as opposed to the “strongly predictive” list) to expand the sensitivity, or coverage of the list. We excluded Vietnamese surnames from this analysis because, unlike the other 5 origin groups, Vietnamese origin was not classified as a “race” in the census years under study.

### Extent to which surname matching assumptions apply to census data on Asian Americans in 1920–1940

#### Family name was accurately recorded as surname in the source data

While some Asian cultural groups use a so-called “Eastern name order,” in which surname precedes individual or given name, name order practices have varied over time and across contexts. For example, Japanese passports used the Eastern order of naming until 1896, when they adopted the Western naming order, which would have been in use in Japanese passports during the period of this study (35). Conversely, Chinese and Korean cultures maintained the practice of listing family names first (35). Meanwhile, though Filipino and South Asian names often followed the “Western order” they may have incorporated multiple family names or surnames, based on maternal maiden names, caste, religion, geography, or honorifics (89, 90). This could lead to incorrect segmentation or transposition of multi-component surnames, as has been observed for two-and three-character Chinese names (91). The discussion section will further elaborate on the potential impact of census enumerators incorrectly entering surnames (Figure 2).

#### Low prevalence of intermarriage with other ethnic or racial groups

Name change at marriage may be less of an obstacle to validity in the present study for a number of reasons. First, name change at marriage is not the norm in all Asian subgroups (35). Furthermore, intermarriage between whites and Asians was legally prohibited by California’s anti-miscegenation law. However, this statute did not regulate Asian interethnic marriages or marriages of Asian individuals to other non-white individuals (92, 93). Some interracial couples likely found ways to circumvent the prohibitions on interracial marriage, but the degree to which these unions occurred is largely unknown (93). Research using 1990 census data identified relatively low rates of out-marriage in Asian immigrant adults aged 65 or older. Among Chinese, Filipino, Indian, Japanese, Korean and Vietnamese men, few had married outside of their own Asian subgroup, with prevalence of out-marriage ranging from 4% of older Chinese men to 12% of older Filipino men. For older married women, the proportions of out-marriage ranged from 6% of Chinese women to 13% of Japanese women and 16% of Korean women. It is thus a reasonable assumption that the prevalence of out-marriage was similarly low in 1920–1940 (94).

#### Second and subsequent generations have similar surnames to those of first-generation immigrants

Although there are certainly cases of Asian immigrants changing or anglicizing surnames after arrival in the United States (95), scholarship suggests that anglicizing names was not as common of an assimilation strategy for Asian immigrants as for some other racialized groups (96). Furthermore, because the surname list comes from administrative data on Asian immigrants, the surname list likely includes anglicized versions of Asian surnames that are prevalent in the immigrant population.

As the source data for the surname list comes 1990, an additional assumption is that an Asian immigrant surname list from 1990 would not be missing important surnames of Asian immigrants and Asian Americans in the early 20th century. We do not have reason to believe that a surname list developed in the 1990 would be inappropriate to apply to populations from the early 20th century. Although the distribution of ethnic subgroups among Asian Americans has shifted over time and early migration from China in the time of the Chinese Exclusion Act often centered around particular clans that shared the same family name (97), a surname list has no indication of the frequency or distribution of specific surnames--it is simply a list of all names, and most names present in the Asian and Asian American population in 1920–1940 would likely still be captured on a surname list in 1990.

#### The population would not contain multiple subgroups with similar surnames

Of the four assumptions, this one is most doubtful when considering Asian Americans in 1920–1940. First, the authors who developed the surname list excluded six surnames (Ha, Jung, Ko, Lee, Lim, Tan) that are common across multiple Asian subgroups and could not reliably predict a specific subgroup. While most subgroups have quite distinctive surnames, Filipinos in California have substantial surname overlap with Latinos due to their common histories of Spanish colonization, which means a criterion based solely on common Filipino names would falsely identify many people of Spanish or Latin American descent. To avoid this, the creators of the unconditional surname list excluded all Filipino names that are on the Spanish surname list from the 1990 census (57), reducing the number of non-Filipinos who are classified as Filipinos, but also missing many Filipinos with Spanish surnames.

### Analysis

All statistical analyses were performed using Stata version 16 (StataCorp LP, College Station, TX). We restricted the analysis to individuals with complete data on assigned race, sex, age, and surname. We calculated descriptive statistics for each census year, presenting frequencies within census year for assigned race, sex, age, and surname categories.

To assess the validity of the surname lists in the census data in each census year, we calculated the sensitivity, specificity, and positive predictive value (PPV) of each surname subgroup classification, using census-designated race as the comparison. Though these measures are often used in clinical settings to quantify the validity of diagnostic tests or screenings, they can also be used to examine the validity of a dichotomous exposure variable (98), such as membership in a racialized group. Sensitivity indicates the proportion of “true positives,” or people in a census racial group whose surname is on the list for that subgroup. Specificity indicates the proportion of “true negatives,” or people who were *not* assigned a given racial group in the census whose surnames were also not on the surname list for that group. Finally, the positive predictive value (PPV) refers to the proportion of people in a surname group who are also assigned that census racial group (71). The PPV is highly variable across populations because it is influenced by the prevalence of the exposure in the population of interest (98). See Table 1 for the formulas used to calculate these probabilities, using the Japanese surname list as an example. See the supplement for the final two by two frequency tables used to calculate these three measures for each of the five surname lists.

**Table 1:** Sensitivity, specificity and positive predictive value of surname lists, illustrated for Japanese surnames.

Surname criteria	Census racial classification		Totals
	Japanese	Not Japanese	
On Japanese list	a	b	a + b
Not on Japanese list	c	d	c + d
Totals	a + c	b + d	N

Sensitivity = P (Japanese surname | Japanese on census) = a/(a + c); Specificity = P (non-Japanese surname | not Japanese) = d/(b + d); PPV = P (Japanese | Japanese surname) = a/(a + b).

Validity calculations require designation of one of the two methods as the “reference standard,” more commonly referred to as the “gold standard.” However, the term “gold standard” implies credibility even if the validity and accuracy of the reference itself is uncertain. Thus, we emulate others in using the more neutral “reference standard” terminology instead (99). We have chosen to label the census racial categories our reference standard, not because we believe it to be theoretically more valid than a surname match, but because an explicit racial classification in a data source is generally used as the default unless it is unavailable. Census racial designations are not objective truths; rather, census enumerators were subject to their own implicit and conscious biases and played active roles in a racial project of categorizing people. We further comment on issues pertaining to use of census designated race as a reference (or “gold”) standard in the discussion section.

We compare the validity measures from the 1920, 1930 and 1940 censuses to those calculated by Lauderdale and Kestenbaum when applying the same unconditional predictive surname classification list to a subsample of the 1990 census.

## Results

We began with the complete count of data for California (*n*_1920_ = 3,433,668, *n*_1930_ = 5,669,757, and *n*_1940_ = 6,879,664), and excluded people missing data on assigned race, age, sex and surname for a final sample of (*n*_1920_ = 3,260,722, *n*_1930_ = 5,317,087, and *n*_1940_ = 6,558,462). Table 2 displays demographic characteristics of California’s population in each decennial census year. California’s population grew substantially between 1920 and 1930. All of the Asian subgroups included in this analysis grew as well, but some did not keep pace with statewide population growth such that their percentage of the total population declined (e.g., from 2.03% Japanese in 1920 to 1.78% in 1930). The Filipino population grew dramatically, from 1,619 in 1920 to 21,099 in 1930. Between 1930 and 1940, the number of people classified as Chinese or Filipino stayed relatively constant, while there were decreases in the number of people classified as “Hindu,” Japanese and Korean. Across successive census years the age distribution of California’s population grew slightly older, and the sex distribution shifted to be more balanced, with a higher proportion of female residents each year. The Asian surname groups are smaller than the corresponding census-assigned race groups for each year.

**Table 2:** Descriptive statistics, complete count decennial census data from California, 1920 (*n* = 3,260,722), 1930 (*n* = 5,317,087), and 1940 (*n* = 6,558,462).

		1920		1930		1940	
		*N*	%	*N*	%	*N*	%
**Census-assigned race**							
Also on surname list	Chinese	22,365	0.69%	31,158	0.59%	33,326	0.51%
	Filipino	1,619	0.05%	21,099	0.40%	21,792	0.33%
	“Hindu”	1,157	0.04%	1,434	0.03%	1,085	0.02%
	Japanese	66,032	2.03%	94,674	1.78%	88,533	1.35%
	Korean	481	0.01%	961	0.02%	853	0.01%
Black		26,691	0.82%	79,532	1.50%	118,279	1.80%
Mexican (1940 only)				147,403	2.77%		
Native American		13,395	0.41%	17,987	0.34%	14,611	0.22%
White		3,103,697	95.18%	4,922,388	92.58%	6,275,818	95.69%
Other		25,285	0.78%	451	0.01%	4,165	0.06%
**Sex**							
Female		1,540,244	47.24%	2,580,287	48.53%	3,227,800	49.22%
Male		1,720,478	52.76%	2,736,800	51.47%	3,330,662	50.78%
**Age**							
<10		520,350	15.96%	782,277	14.71%	809,042	12.34%
10–19		476,486	14.61%	790,323	14.86%	941,720	14.36%
20–29		553,407	16.97%	899,776	16.92%	1,130,214	17.23%
30–39		589,195	18.07%	915,891	17.23%	1,087,279	16.58%
40–49		476,037	14.60%	800,037	15.05%	967,673	14.75%
50–59		331,191	10.16%	567,971	10.68%	783,747	11.95%
60–69		197,069	6.04%	354,776	6.67%	520,257	7.93%
70+		116,987	3.59%	206,036	3.87%	318,530	4.86%
**Surname category**							
Chinese		15,294	0.47%	22,337	0.42%	24,863	0.38%
Filipino		1,262	0.04%	6,346	0.12%	7,922	0.12%
Indian		938	0.03%	1,314	0.02%	1,145	0.02%
Japanese		35,695	1.09%	58,964	1.11%	54,984	0.84%
Korean		1,358	0.04%	1,635	0.03%	1945	0.03%

Table 3 presents the sensitivity, specificity and PPV comparing the two classification approaches for each Asian subgroup, by census year. We also include sensitivity and PPV from comparing the surname list with 1990 census data, published elsewhere (70).

**Table 3:** Sensitivity, specificity, and positive predictive value of 5 surname lists compared to census racial classification using all records from California, 1920–1940, with comparison to validity statistics comparing surname lists to the 1990 census, published elsewhere (70).

Surname subgroup	Census year	Comparison to identification by census race variable		
		Sensitivity	Specificity	PPV
Chinese	1920	0.60	>0.99	0.87
	1930	0.67	>0.99	0.93
	1940	0.65	>0.99	0.88
	(c.f. 1990)	0.70	(n.a.)	0.76
Filipino	1920	0.10	>0.99	0.13
	1930	0.21	>0.99	0.68
	1940	0.21	>0.99	0.56
	(c.f. 1990)	0.29	(n.a.)	0.86
Indian	1920	0.54	>0.99	0.67
	1930	0.61	>0.99	0.66
	1940	0.56	>0.99	0.53
	(c.f. 1990)	0.38	(n.a.)	0.77
Japanese	1920	0.51	>0.99	0.94
	1930	0.62	>0.99	0.99
	1940	0.59	>0.99	0.95
	(c.f. 1990)	0.71	(n.a.)	0.92
Korean	1920	0.44	>0.99	0.16
	1930	0.40	>0.99	0.23
	1940	0.45	>0.99	0.20
	(c.f. 1990)	0.54	(n.a.)	0.81

### Sensitivity

The subgroups for whom surname criteria have the highest sensitivity in 1920–1940 are Chinese (ranging from 0.60 to 0.67 across census years), followed by Indian (0.54–0.61) and Japanese (0.51–0.62). Sensitivity was much lower for Korean (0.40–0.45) and Filipino (0.10–0.21) surnames.

With the exception of Indian surnames, the sensitivities of surname criteria are lower in 1920–1940 census data than in the 1990 census. The extent of the difference varies by subgroup; the sensitivity of the Chinese surname criteria in 1930 (0.67) is not far from the sensitivity in 1990 (0.70). By contrast, the sensitivity of Japanese surname criteria throughout 1920–1940 is substantially lower than in 1990. The sensitivity of Indian surname criteria was substantially higher in 1920–1940 (0.54–0.61) than in 1990 (0.38).

Trends in sensitivity across census years also vary by subgroup. Chinese, Japanese and Indian surname criteria were most sensitive in the 1930 census compared to 1920 and 1940, whereas Korean surname criteria were the lowest in the 1930 census. The sensitivity of Filipino surname criteria was extremely low in 1920 (0.10) and remained steady from 1930 to 1940 (0.21 in each year).

### Specificity

Specificity exceeded 0.99 for all surname lists across all census years.

### Positive predictive value

PPV for surname criteria varied widely between subgroups and across census years. Japanese surnames had the highest PPV (ranging from 0.94 to 0.99), followed by Chinese surnames (0.87–0.93). Indian surnames had PPV ranging from 0.53 to 0.67. Korean surnames had the lowest PPV (0.16–0.23). The PPV of Filipino surnames increased substantially from 1920 (0.13) to 1930 (0.68) and 1940 (0.56).

For Japanese and Chinese surnames, the PPV of surname criteria in the 1920–1940 census are higher than in the 1990 census. PPV of Indian surnames in 1920–1940 are slightly lower than in 1990. By contrast, the PPV of Korean surnames throughout 1920–1940 is substantially lower than in 1990. Filipino surnames have extremely low PPV (0.13) in 1920 and increase in 1930–1940 but are still lower than in 1990.

## Discussion

This paper examined the effectiveness of using surnames to classify Chinese, Indian, Japanese, Korean and Filipino subgroups in census data from 1920 to 1940. We found remarkably lower agreement between surname category and census-designated race in 1920–1940 compared to the application of the same surname criteria in 1990.

Sensitivity, or (proportion of “true positives”) indicates the proportion of people in a census racial group whose surname is on the list for that subgroup. Surname criteria identified more than half of people assigned to the Chinese, Indian and Japanese census racial groups across census years 1920–1940. However, the Chinese and Japanese surname list identified a lower proportion of people classified in those racial groups than when the same lists were used with the 1990 census. The sensitivity of the Korean surname list was lower, identifying 40–45% of people the census categorized as Korean. The sensitivity of the Filipino list was even lower, only identifying 10–21% of people assigned Filipino race on the census.

All surname lists had specificity (proportion of “true negatives”) greater than 99%, meaning that nearly all the people who were *not* assigned a given racial group were also not on the surname list for that group. Fewer than 1 % of people were falsely identified through surname criteria for that group.

Positive predictive value (PPV, proportion of people in a surname group who are also assigned that census racial group) varied widely across subgroups and census years. While sensitivity and specificity describe the validity of a classification system itself, PPV varies with the population prevalence of the characteristic being measured. This explains much of the variation in PPV in historical census data compared to the 1990 census comparison. For example, the low PPV of the Korean surname list in 1920–1940 corresponds to the much smaller Korean population during those years compared to 1990. By contrast, Chinese and Japanese groups were a *larger* proportion of the California population in 1920–1940 than the total United States population in 1990. The Filipino population grew dramatically between the 1910 and 1920 censuses; as expected the PPV increased in turn.

Generally speaking, operationalizing Asian racial subgroups using surname underestimates the size of the groups in historical census data, but minimally misclassifies non-Asian people as members of Asian subgroups. As expected, Filipino surname criteria had the lowest sensitivity of the five subgroups in 1920–1940 and 1990. Korean surnames had very low positive predictive value in 1920–1940. Overall, this raises caution about the use of validated Asian surname criteria as a proxy for racial origin in historical data, particularly for people of Filipino and Korean descent.

### Limitations

One key limitation of our study is that our calculation of validity measures demonstrates the level of agreement between the two classification methods but does not reveal whether they are statistically different because each comparison examines a separate dichotomous variable (i.e., Japanese and non-Japanese as defined by each of the two methods) rather than a complete racial distribution. While the Census racial designation dichotomous variables are drawn from a categorical racial distribution, the surname method does not easily generate such a distribution. The creators of these surname lists caution against using a combination of the lists to identify an overall “Asian-American” group as it would lead to overrepresentation of surname groups whose lists have higher sensitivity (70). Future research could attempt to adjust for the different sensitivities of each surname list to enable a formal statistical comparison of categorical racial distributions from surname lists and census racial classifications.

Another limitation of this study is that we were unable to quantify potential error in the census dataset or fully account for the impact of this error on our validity measures. While errors introduced during the original enumeration might be of interest to researchers in and of themselves, digitization or indexing introduces another layer of error. Transcription errors of Asian surnames at both stages of dataset creation remain underexamined in the literature. One study comparing two independent transcriptions of the 1940 census found for individuals born in England (chosen to represent the English-speaking foreign-born population) versus those born in Italy (chosen to represent the non-English-speaking foreign-born population) both first name (7.2% vs. 14.3%) and surname transcriptions (17.0% vs. 31.5%) disagreed almost twice as often for those born in Italy (100). We found only one paper that considers transposition of family name and individual name for an Asian subgroup specifically (91). Postel identifies three types of issues commonly found in the recording of Chinese names: segmentation, name order, and standardization. These types of mistakes were geographically and temporally inconsistent across enumeration contexts. For example, segmentation errors during indexing led 79% of Chinese individuals to have either their first or last name missing because all the components of their name were allocated to a single variable rather than being split into a personal name and a family name (91). All of these factors could undermine our assumption that family names were accurately recorded under surname for Chinese immigrants.

Finally, an important consideration and caveat when comparing validity statistics across census years is that the reference standard, census racial classification, is far from a gold standard, and certainly varied substantially in its accuracy in 1920–1940, when census enumerators assigned race, compared to 1990 when race was supposed to be self-identified. Our study did not account for the enumerator bias present within the census racial classifications themselves, but the work of other scholars (9, 101, 102) can serve as a guide to future efforts to quantify bias in census racial classifications.

As such, differences in validity statistics may reflect inadequacies of census racial classification as well as the appropriateness of surname classification. With this caveat in mind, we believe that the factors contributing to the lower validity of the surname criteria found in our analysis are many and complex, and thus need additional research to extricate. The following section highlights some possible explanations for the lower validity, each of which represents a promising path for future improvement of the use of surname criteria, census racial classifications, or both.

### Possible explanations for disagreement between surname criteria and census classifications

Based on the historical research and limited quantitative analysis of the dynamics at play in the changes in surname patterns and the assignment of race during census enumeration, we can speculate about a few possible factors. Census enumeration instructions from each decade reinforce the agency given to individual enumerators in assigning race, even within the bounds of their official instructions and training, as well as challenges they faced in their task. The Census Bureau did not prioritize use of translators and instead relied upon the unpaid translation work of family members or neighbors (49–51). Census workers collecting information from more recent immigrants were attempting to communicate with people who may have spoken an unfamiliar language with an unfamiliar alphabet, which likely produced errors in both the spelling and romanization of surname and the categorization of race. These communication barriers could further interact with imbalanced power dynamics in a variety of congregate living settings, with foremen or institutional authorities of a different race making decisions about racial classification and spelling of names even further removed from the individual being described than in a typical enumerator observation.

Beyond the impact of language barriers, both the implicit and conscious biases of census enumerators likely impacted their assignment of race to the individuals they enumerated. Velyvis et al. and Loveman present a compelling analysis of changing racial boundaries in the 1910 and 1920 censuses in United States occupied Puerto Rico (101, 102). They provide evidence that census instructions and procedures sometimes conflicted with enumerators’ socially-defined conceptualization of race based on appearance or phenotype and emphasize the active role census enumerators played in this act of racialization. They encountered thousands of instances across both censuses where a small group of census supervisors “corrected” the racial categorizations in post-enumeration edits of the census broadsheets. These edits to an individual’s race were usually performed on the basis of parental race or similar rules of racial heritability or “racial logic,” suggesting contested racialization processes and a degree of error inherent in attempting to impose simplistic logic onto ambiguous sociopolitical categories (101, 102). While the Asian population of Puerto Rico was small and thus did not feature in their commentary, it stands to reason that the enumerators hired and trained under the same federal agency, the United States Census Bureau, were able to play similarly active roles in the racialization of the people they enumerated, albeit under a different regional and sociopolitical context. It is unclear whether a similar editing process took place in the California censuses, but investigating the original census broadsheets could be a rich avenue for future study if data use agreements allow.

Shah presents an earlier relevant example of the role of enumerator racial bias—specifically anti-Chinese bias—on census data collection. In the 1870 United States census, two census enumerators with known biographical information produced vastly different counts of the number of Chinese women with an occupation of “prostitute” in their respective enumeration districts in San Francisco’s Chinatown. One enumerator often divided the Chinese residents in congregate living situations into two families by sex and listed the occupation of all the men as “laborer” and all the women as “prostitute.” This resulted in 90% of Chinese women over the age of 12 being designated prostitutes in his district. The other enumerator, who was more sympathetic to Chinese immigrants, recognized more complex family delineations and only designated 53% of Chinese women over the age of 12 as prostitutes (9).

As briefly mentioned in the introduction, ethnic and regional diversity within the Asian countries of origin may contribute to the lack of agreement between census racial categorization and surname match. In the age of Chinese exclusion, which covers the entirety of our study period, Chinese immigrants navigated a complex array of United States immigration and naturalization laws. Thus, continued migration, though occurring at lower numbers than before, was often facilitated by clan (i.e., surname) associations of Chinese immigrants and their American-born children already in the United States (97). While the sensitivity of the Chinese surname list as applied in our analysis was comparable to the sensitivity reported by Lauderdale and Kestenbaum (70), the contextual knowledge of prevalence of specific Chinese surnames could perhaps be used to further improve sensitivity in future applications. In contrast, our sensitivity values for the Japanese and Filipino surname lists were much lower overall than those reported by Lauderdale and Kestenbaum. There is also evidence of regional emigration patterns and labor recruitment practices in Japan and the Philippines (103, 104) and migrants from these regions could have had distinct surname patterns that changed more over time than Chinese surname distributions.

Global power structures, especially in an age of imperialism, had a significant impact on surnames in certain contexts. Of particular note, our period of study coincides with periods of colonial oppression of two national origin groups: the Japanese occupation of Korea from 1910 to 1945 and American control of the Philippines from the late 1890s to 1934. In 1939 the Japanese government enacted legislation pressuring Koreans to assimilate to Japanese society by changing their surnames, resulting in many ethnic Koreans possessing Japanese surnames in the later period of Japanese occupation (104). This practice likely occurred too late to affect Korean immigrants or Korean-Americans in our study population; however, some scholars claim this practice began earlier (105), both involuntarily and voluntarily, with some upper-class Koreans adopting Japanese surnames to increase social status (106). Furthermore, Korea had only recently attained independence from Chinese rule in the late 1890s, so the influence of Chinese rule on surnames likely persisted as well (106). Unlike in the case of shifting political boundaries in Europe (e.g., German Poland, Russian Poland, Alsace-Lorraine, Bavaria etc. in 1920), the census enumeration instructions did not specify how the Japanese occupation of Korea would affect the recording of race or birthplace for either Japanese or Korean individuals (49–51).

The United States occupation of the Philippines followed several centuries of Spanish colonization of the archipelago. Early Filipino immigrants to the United States were largely *pensionados*, or government-sponsored scholarship students from upper-class Filipino families, and self-supporting students from middle-class families. The drastic increase in the number of Filipinos in the United States through the 1920s (especially after the Immigration Quota Act of 1924 barred immigration from other Asian countries that had previously provided a steady source of immigrant workers) was driven primarily by laborers (10). If this shift in socioeconomic status of Filipino immigrants manifested in differential surname patterns, it may have contributed to the jump in sensitivity from 10 to 21% between the 1920 and 1930 censuses.

## Implications and conclusion

This paper adds to the literature by extending Asian surname criteria matching to historical data. While Asian surnames have been used in multiple health studies (86–88), we encountered only a few examples of applying surname criteria in the context of historical research or historical health research (107–111). Spanish surname lists have been used extensively, but the potential to identify Asian persons in data sources without information on race, and to differentiate among Asian subgroups within historical data sources with less specific racial classifications remains relatively untapped.

Historical data sources present rich opportunities to document and analyze dynamics of anti-Asian racism that underpin current inequities. Historical events still affect contemporary health outcomes, whether they manifest through intergenerational trauma or in the biases of the very data relied upon for longitudinally assessing population-level health (13). Public health scholars can heed calls to examine our history in order to understand and dismantle contemporary injustices (11–13). A growing literature uses historical data to examine structural drivers of Black-white racial inequalities in health (11, 112–115), but research extending this approach to other racialized groups is limited, partly because of the inconsistency or unavailability of historical data on these populations.

The lower level of agreement between the surname-criteria and census designation in measuring race does not mean the data are not useful or valid, only that one method may be more valid for specific research questions and that each has its own limitations that should be accounted for in discussing results. In fact, some of the possible biases in the census racial data and their effects on the dataset pose interesting research questions in and of themselves. Additional research could explore multifactor measures of race and ethnicity (17, 20) and explicitly test the underlying assumptions of surname analysis. Class-based paradigms of race (29) suggest occupation in the context of exploitative labor practices could be one census variable used in such a multifactor measure. Molina’s analysis of discrimination against Chinese launderers in the name of “public health” in early 1900s Los Angeles further supports this suggestion (47). Quantitative researchers may shy away from the complexity of conducting historical research about racism and health, but we hope this study exemplifies how variables can be used thoughtfully and contextually while still producing categories feasible for analysis. Demography and statistics were once used by white supremacists and eugenicists as tools to “prove” the biological inferiority of non-white people. The census was not only used in service of racist research, but was in turn shaped by the research goals of those same political actors. Unless we adequately interrogate our usage of this same data, we risk reproducing the harm of racist power structures.

## Data availability statement

The data analyzed in this study is subject to the following licenses/restrictions: Researchers can access restricted complete count data (including names and string variables) for United States censuses 1870–1940 through a research agreement with IPUMS United States. Requests to access these datasets should be directed to ipums@umn.edu.

## Author contributions

MK, NN, and SG conceptualized the study and developed the methodology. MK developed the background and theory in consultation with MK, NN, SG, SH, and AS. MK and SG cleaned the data. SG and NN conducted the statistical analysis. MK and NN prepared the manuscript draft. All authors contributed to the article and approved the submitted version.

## Funding

The authors gratefully acknowledge the National Human Genome Research Institute (grant R01 HG010567-05) for funding support.

## Conflict of interest

The authors declare that the research was conducted in the absence of any commercial or financial relationships that could be construed as a potential conflict of interest.

## Publisher’s note

All claims expressed in this article are solely those of the authors and do not necessarily represent those of their affiliated organizations, or those of the publisher, the editors and the reviewers. Any product that may be evaluated in this article, or claim that may be made by its manufacturer, is not guaranteed or endorsed by the publisher.
