# Supplementary material for: Operationalizing racialized exposures in historical research on anti-Asian racism and health: a comparison of two methods
Source: Front Public Health. 2023 Jul 6;11:983434. doi: 10.3389/fpubh.2023.983434 (PMC10359498; doi:10.3389/fpubh.2023.983434)
Supplement: Supplementary file 1 [file Table_1.DOCX]

**Sensitivity, specificity and positive predictive value of Chinese surname list compared to census racial classification using all records from California, 1920-1940, with comparison to validity statistics comparing Chinese surname lists to the 1990 census, published elsewhere (Lauderdale and Kestenbaum, 2000).**

| **Surname criteria** | **1920 Census racial classification** | | **Totals** |
| --- | --- | --- | --- |
|  | **Chinese** | **not Chinese** |  |
| **On Chinese list** | 13,314 | 1,980 | 15,294 |
| **not on Chinese list** | 9,051 | 3,236,377 | 3,245,428 |
| **Totals** | 22,365 | 3,238,357 | 3,260,722 |

| **Surname criteria** | **1930 Census racial classification** | | **Totals** |
| --- | --- | --- | --- |
|  | **Chinese** | **not Chinese** |  |
| **On Chinese list** | 20,791 | 1,546 | 22,337 |
| **not on Chinese list** | 10,367 | 5,284,383 | 5,294,750 |
| **Totals** | 31,158 | 5,285,929 | 5,317,087 |

| **Surname criteria** | **1940 Census racial classification** | | **Totals** |
| --- | --- | --- | --- |
|  | **Chinese** | **not Chinese** |  |
| **On Chinese list** | 21,825 | 3,038 | 24,863 |
| **not on Chinese list** | 11,501 | 6,522,098 | 6,533,599 |
| **Totals** | 33,326 | 6,525,136 | 6,558,462 |

**Sensitivity, specificity and positive predictive value of Filipino surname list compared to census racial classification using all records from California, 1920-1940, with comparison to validity statistics comparing Filipino surname lists to the 1990 census, published elsewhere (Lauderdale and Kestenbaum, 2000).**

| **Surname criteria** | **1920 Census racial classification** | | **Totals** |
| --- | --- | --- | --- |
|  | **Filipino** | **not Filipino** |  |
| **On Filipino list** | 163 | 1,099 | 1,262 |
| **not on Filipino list** | 1,456 | 3,258,004 | 3,259,460 |
| **Totals** | 1,619 | 3,259,103 | 3,260,722 |

| **Surname criteria** | **1930 Census racial classification** | | **Totals** |
| --- | --- | --- | --- |
|  | **Filipino** | **not Filipino** |  |
| **On Filipino list** | 4,340 | 2,006 | 6,346 |
| **not on Filipino list** | 16,759 | 5,293,982 | 5,310,741 |
| **Totals** | 21,099 | 5,295,988 | 5,317,087 |

| **Surname criteria** | **1940 Census racial classification** | | **Totals** |
| --- | --- | --- | --- |
|  | **Filipino** | **not Filipino** |  |
| **On Filipino list** | 4,438 | 3,484 | 7,922 |
| **not on Filipino list** | 17,018 | 6,533,522 | 6,550,540 |
| **Totals** | 21,456 | 6,537,006 | 6,558,462 |

**Sensitivity, specificity and positive predictive value of Indian surname list compared to census racial classification using all records from California, 1920-1940, with comparison to validity statistics comparing Indian surname lists to the 1990 census, published elsewhere (Lauderdale and Kestenbaum, 2000).**

| **Surname criteria** | **1920 Census racial classification** | | **Totals** |
| --- | --- | --- | --- |
|  | **Indian** | **not Indian** |  |
| **On Indian list** | 625 | 313 | 938 |
| **not on Indian list** | 532 | 3,259,252 | 3,259,784 |
| **Totals** | 1,157 | 3,259,565 | 3,260,722 |

| **Surname criteria** | **1930 Census racial classification** | | **Totals** |
| --- | --- | --- | --- |
|  | **Indian** | **not Indian** |  |
| **On Indian list** | 870 | 444 | 1,314 |
| **not on Indian list** | 564 | 5,315,209 | 5,315,773 |
| **Totals** | 1,434 | 5,315,653 | 5,317,087 |

| **Surname criteria** | **1940 Census racial classification** | | **Totals** |
| --- | --- | --- | --- |
|  | **Indian** | **not Indian** |  |
| **On Indian list** | 604 | 541 | 1,145 |
| **not on Indian list** | 481 | 6,556,836 | 6,557,317 |
| **Totals** | 1,085 | 6,557,377 | 6,558,462 |

**Sensitivity, specificity and positive predictive value of Japanese surname list compared to census racial classification using all records from California, 1920-1940, with comparison to validity statistics comparing Japanese surname lists to the 1990 census, published elsewhere (Lauderdale and Kestenbaum, 2000).**

| **Surname criteria** | **1920 Census racial classification** | | **Totals** |
| --- | --- | --- | --- |
|  | **Japanese** | **not Japanese** |  |
| **On Japanese list** | 33,650 | 2,045 | 35,695 |
| **not on Japanese list** | 32,382 | 3,192,645 | 3,225,027 |
| **Totals** | 66,032 | 3,194,690 | 3,260,722 |

| **Surname criteria** | **1930 Census racial classification** | | **Totals** |
| --- | --- | --- | --- |
|  | **Japanese** | **not Japanese** |  |
| **On Japanese list** | 58,346 | 618 | 58,964 |
| **not on Japanese list** | 36,328 | 5,221,795 | 5,258,123 |
| **Totals** | 94,674 | 5,222,413 | 5,317,087 |

| **Surname criteria** | **1940 Census racial classification** | | **Totals** |
| --- | --- | --- | --- |
|  | **Japanese** | **not Japanese** |  |
| **On Japanese list** | 52,043 | 2,941 | 54,984 |
| **not on Japanese list** | 36,490 | 6,466,988 | 6,503,478 |
| **Totals** | 88,533 | 6,469,929 | 6,558,462 |

**Sensitivity, specificity and positive predictive value of Korean surname list compared to census racial classification using all records from California, 1920-1940, with comparison to validity statistics comparing Korean surname lists to the 1990 census, published elsewhere (Lauderdale and Kestenbaum, 2000).**

| **Surname criteria** | **1920 Census racial classification** | | **Totals** |
| --- | --- | --- | --- |
|  | **Korean** | **not Korean** |  |
| **On Korean list** | 214 | 1,144 | 1,358 |
| **not on Korean list** | 267 | 3,259,097 | 3,259,364 |
| **Totals** | 481 | 3,260,241 | 3,260,722 |

| **Surname criteria** | **1930 Census racial classification** | | **Totals** |
| --- | --- | --- | --- |
|  | **Korean** | **not Korean** |  |
| **On Korean list** | 380 | 1,255 | 1,635 |
| **not on Korean list** | 581 | 5,314,871 | 5,315,452 |
| **Totals** | 961 | 5,316,126 | 5,317,087 |

| **Surname criteria** | **1940 Census racial classification** | | **Totals** |
| --- | --- | --- | --- |
|  | **Korean** | **not Korean** |  |
| **On Korean list** | 384 | 1,561 | 1,945 |
| **not on Korean list** | 469 | 6,556,048 | 6,556,517 |
| **Totals** | 853 | 6,557,609 | 6,558,462 |
